# Effect of pretreatment and temperature on the drying kinetics and physicochemical and techno-functional characteristics of pumpkin (*Cucurbita maxima*)

**DOI:** 10.1016/j.heliyon.2021.e06802

**Published:** 2021-04-16

**Authors:** Carlos J. Márquez-Cardozo, Birina L. Caballero-Gutiérrez, Héctor J. Ciro-Velázquez, Diego A. Restrepo-Molina

**Affiliations:** Universidad Nacional de Colombia, Facultad de Ciencias Agrarias, Departamento de Ingeniería Agrícola y Alimentos, Medellín, Colombia

**Keywords:** Activation energy, Bioactive compound, Powder, Vegetables

## Abstract

The study was carried out to assess fresh slices and thermally pretreated pumpkin (*Cucurbita maxima*) dried at temperatures of 55 °C, 60 °C, 65 °C, and 70 °C. The drying kinetics and quality attributes of the dried product were determined, and results indicated that the modified Page model was the best fit, with activation energies of 29.47 kJ mol^−1^ and 16.06 kJ mol^−1^ for drying fresh and thermally pretreated slices, respectively. A significant effect (p < 0.05) related to thermal pretreatment and temperature was evidenced on the physicochemical properties. The fresh pulp powders presented the following ranges of moisture and color (ΔE), 7.10%–8.31% w.b.; 21.23–25.23, respectively, and for the pretreated pulp powders, they were 8.94%–11.54% w.b., and from 19.00- 28.30, respectively. There were no significant effects on the techno-functional properties in the powders; cold water solubility was 5.36%–6.46%, water absorption capacity was 3.42–6.52 g/g, and oil absorption capacity was 1.00–1.30 g/g. The carbohydrate and fiber contents significantly decreased in the pretreated powder. An increase in antioxidant activity was found in fresh and thermally pretreated pulp powder at a temperature of 70 °C, presenting values between 2.23-2.98 μmol Trolox equivalent g^−1^d.b. evaluated by the DPPH method and between 40.48-45.92 μmol Trolox equivalent g^−1^d.b. by ABTS, and no significant differences (p > 0.05) were determined after pulp pretreatment. The total content of carotenoids presented retention percentages for fresh pulp powders of 52.09%, 41.92%, 30.55%, and 22.79%, while for pretreated pulp powders, they were 30.67%, 32.86%, 24.84%, and 14.71% when dried at temperatures of 55 °C, 60 °C, 65 °C, and 70 °C, respectively. The powders obtained from heat-pretreated pumpkin pulp showed significant differences (p < 0.05) in physicochemical characteristics and total carotenoids, but they were not found (p > 0.05) in the techno-functional properties and antioxidant activity evaluated by the DPPH and ABTS methods.

## 1 Introduction

Pumpkin, also known as auyama, squash, or sambo, belongs to the family of cucurbits and is classified into the species *Cucurbita maxima, Cucurbita pepo, Cucurbita moschata,* and *Cucurbita mixta;* it is native to South America, where it grows wild on the southern cone and Mesoamerica (Lorello et al., 2016). Worldwide production is approximately 27643932 t, and the harvested area is 2042955 ha with an average yield of 13.53 t/ha (FAO, 2018). In Colombia, the harvested area is close to 6620 ha, with a production of 92180 metric tons and an average yield of 13.92 t/ha. In the last five years, the production of this crop has increased by approximately 80% and is considered a product with high economic importance in the rural sectors of Colombia (Agronet, 2017).

Its physical structure is a large berry with a hard shell and fleshy pulp, yellow-orange in color, dense, with a firm texture and sweet flavor, particularly striking for different gastronomic preparations; from a nutritional point of view, it has low concentrations of carbohydrates (8.8%) compared to other vegetables, protein around 1%, fat near 0.5%, and fiber at 1%. Pumpkin is also rich in minerals such as potassium (439 mg), calcium (26 mg), and phosphorus (17 mg) (Jaeger et al., 2012; Mi et al., 2012; Rodríguez et al., 2018). In addition, in its bright orange flesh, it has high concentrations of β-carotene, an important precursor of vitamin A (Amorim et al., 2014; Saini et al., 2015; Rodríguez et al., 2018), reporting total carotenoids in *C. moschata* pulp between 160 μg/g d.b. and 1399 μg/g d.b. (Jacobo et al., 2011), and 4.58 ± 2.27 mg/100 d.b. of β-carotenes in *C. pepo,* and 2.92 ± 1.40 mg/100 d.b. in *C. moschata* (Kulczynski and Gramza, 2019). It is also rich in phenolic compounds, 476 mg equivalent gallic acid/100 g d.b (Jacobo et al., 2011), and 2292 ± 1.1 mg equivalent gallic acid/100 g d.b. (Bahramsoltani et al., 2017).

Dehydration processes in fruits and vegetables prolong their shelf life, reducing water content and enzymatic and microbiological activity, but they can significantly affect bioactive compounds such as vitamins, antioxidant activity, and phenolic compounds. Pretreatment carried out before drying could also influence the concentrations of these compounds, possibly due to structural changes in the food that facilitate the dehydration process as well as enzymatic inactivation (Morais et al., 2018). The combination of thermal pretreatment of the product and drying air temperature has shown an important effect on the drying time and organoleptic and quality properties of the final product (Gazor et al., 2014). Some studies report that dehydration by forced convection facilitates the use of this product as a functional and nutritional alternative in the inclusion of food matrices, such as bakery products, dairy foods (yogurt, ice cream, flavored milk), soups, sauces, and baby foods (Gutiérrez, 2018).

In the dehydration of fruits and vegetables, improving the quality of the final product, minimizing the effect on nutritional characteristics, and lowering the energy consumption of the process are important aspects to be considered. Thus, mathematical models of the drying curves allow for the estimated time required to reduce the amount of water in the product under different conditions, improving the process efficiency (Purlis, 2019), and together with the quality characteristics of the product, allow for the best conditions of the drying process (Andrade et al., 2011). Several mathematical models that have been used in thin layers can be used to describe the drying kinetics in food (Gómez et al., 2019; Komolafe et al., 2019).

The aim of this study was to evaluate the effect of pretreatment via thermal heating on pumpkin pulp slices (*C. maxima*) and compare them with fresh pulp slices that had been dehydrated by forced convection, and then the physicochemical, techno-functional, and functional attributes of the powders were evaluated.

## 2 Materials and methods

### 2.1 Materials

Pumpkins (*Cucurbita maxima*) were harvested in the Municipality of Dabeiba (Antioquia, Colombia), stored under ambient temperature conditions (23 °C and 65% RH), washed, and disinfected with 50 ppm sodium hypochlorite solutions. The epidermis and peduncles were removed from the fruits; the pulp was cut into slices 5 ± 1.0 mm thick, obtaining experimental units (EU) of 1.5 ± 0.5 kg for fresh pulp treatments. The same procedure was performed in the experimental units (EU), which were subjected to thermal heating for 5 min while maintaining temperature at 90 °C.

### 2.2 Drying process

Fresh pulp- and heat-pretreated slices of pumpkin (*Cucurbita maxima*) were subjected to convective drying using temperatures of 55 °C, 60 °C, 65 °C, and 70 °C and an air velocity of 3.7 ms^−1^ in Memmert Universal Oven UF750 drying equipment. Samples were removed from the dryer once they reached the equilibrium moisture content (Giraldo et al., 2010). The initial and final moisture contents were determined in an oven at 105 °C according to AOAC (1990). To obtain the drying curve, pumpkin samples were weighed in triplicate for each hour of drying. Once the pumpkin pulp slices were dried through the different treatments until reaching the equilibrium moisture content, they were subjected to grinding (IMA model MOL10) and sieved in a 100 mesh (0.149 mm).

### 2.3 Drying kinetics

The moisture ratio (MR) was calculated using Eq. (1), where $\;M_{t}$, $M_{0}$, and $M_{e}$ represent the moisture content at any time of drying, initial moisture content, and equilibrium moisture content, respectively (Gómez et al., 2019; Komolafe et al., 2019).

(1) $$\text{MR}=\frac{{\text{M}}_{\text{t}}-{\text{M}}_{\text{e}}}{{\text{M}}_{\text{i}}-{\text{M}}_{\text{e}}}$$

Seven mathematical models in the thin layer were used to represent the drying kinetics were calculated according to Eqs. (2), (3), (4), (5), (6), (7) and (8) according to Table 1 (Komolafe et al., 2019).

**Table 1:** Mathematical models of thin layer drying.

Model	Equation	
Newton	MR=exp(−kt)	(2)
Page	$\text{MR}=\text{exp}\left(-{\text{kt}}^{\text{n}}\right)$	(3)
Modified Page	$\text{MR}=\text{exp}\left[-{\left(\text{kt}\right)}^{\text{n}}\right]$	(4)
Henderson and Pabis	MR=aexp(−kt)	(5)
Modified Henderson and Pabis	MR=a exp(−kt)+b exp(−gt)+c exp(−ht)	(6)
Logarithmic	MR=a exp(−kt)+c	(7)
Midilli et al.	$\text{MR}=\text{a}\;\text{exp}\left(-{\text{kt}}^{\text{n}}\right)+\text{bt}$	(8)

The parameters (k, a, b, c, g, h and n) of the models given in Table 1 were estimated by regression using DATAFIT software, version 9.1.32 (Oakdale Engineering). The determination coefficient (R^2^), reduced chi-squared ($x^{2}$) Eq. (9), and root mean square error (R_MSE_) Eq. (10) were defined for each model. For a good fit, the values of R^2^ must be the greatest, and the $x^{2}$ and R_MSE_ values must be the lowest (Largo et al., 2014).

(9) $$x^{2}=\left(\frac{\sum_{i=1}^{N}{\left(M_{R,exp,i}-M_{R,pre,i\;}\right)}^{2}}{\text{N}-\text{z}}\right)$$

(10) $$R_{MSE}={\left[\frac{1}{N}\;\overset{N}{\underset{i=1}{\sum }}{\left(M_{R,exp,i}-M_{R,pre,i\;}\right)}^{2}\right]}^{1/2}$$

where M_R,exp,i_ and M_R,pre,i_ are experimental data, N is the number of observations, and z is the number of constants for each model.

The activation energy (E_a_) was calculated to be the best drying mathematical model found according to the established statistical parameter and calculated as follows Eq. (11).

(11) $$\text{k}=A\;\exp\left(\frac{-Ea}{RT}\right)$$

In this expression, R is the universal gas constant (8.3143 J K^−1^·mol^−1^), T is the temperature in Kelvin, A is the constant of the equation, and “k” is the response variable (the drying constant).

### 2.4 Quality attributes of pumpkin powder

For the final powder product, the following parameters were determined: moisture content according to the official oven gravimetric method AOAC (1990) expressed as a percentage on a wet basis (w.b.), water activity (a_w_) using a water activity meter (Aqualab 3 TE) at a temperature of 25 °C, color properties by the CIE-L^∗^a^∗^b^∗^ method using an X-Rite sphere spectrophotometer (SP-60, with a 4 mm aperture, D-65 illuminant and 2° standard observer). In addition, the total color change (ΔE) was calculated with Eq. (12), where L_0_, a_0_, and b_0_ are the initial conditions of the fresh pulp.

(12) ΔE=((L0−L)2+(a0−a)2+(b0−b)2)12

The content protein analysis was evaluated by the Kjeldahl volumetric method (AOAC 955.04/90), the ash content by a gravimetric method (AOAC 923.03/90), the fat content by a Soxhlet extraction method (AOAC 920.39/90), and total dietary fiber (AOAC 985.29/90). In addition, the carbohydrate content in the fruit differed, both for fresh and precooked pumpkin powders expressed in g/100 g on a wet basis. Additionally, the techno-functional properties of pumpkin powder, such as the cold water solubility (CWS) are expressed as a percentage, the water absorption capacity (WAC) and oil absorption capacity (OAC) are indicated in g/g sample, and bulk density (BD) (g/mL), were quantified according to the methodology proposed by Salcedo et al. (2017).

### 2.5 Antioxidant activity by DPPH, ABTS, and total carotenoid content

For sample preparation to determine antioxidant activity by the DPPH and ABTS methods, 10 mg of pumpkin powder was weighed and homogenized with water and ethanol (1:1) with an IKA T25 ultraturrax in 10 mL of solvent mixtures. For the quantification of total carotenoids, 30 mg of powder was weighed with 10 mL of acetone solution (Merck), stored at 4 °C for 30 min, shaken in a Fisher Scientific vortex mixer and centrifuged at 4000 rpm for 10 min. With the fresh and heat pretreated pulp, 3 g was weighed and mixed with 4 mL of acetone, the supernatant was collected in a tube until 20 mL of solution was obtained, and the procedure described above was applied.

For the determination of the antioxidant activity by DPPH (2,2-diphenyl-1-picril-hydrazyl), the method described by Brand-Williams et al. (1995) and modified by Zapata et al. (2017) was used, where 10 μL aliquots of the extract were placed in an Eppendorf microtube, and 990 μL of DPPH radical was added. The samples were kept at room temperature for 30 min in the dark, and their absorbance at 517 nm was measured. Three extractions were performed for each treatment trial. The results are expressed as μmol Trolox equivalent g^−1^d.b. The antioxidant activity determined by ABTS^•+^ (2,2′-azino-bis-3-ethylbenzothiazoline-6-sulfonic) was performed according to the modified methodology by Londoño et al. (2017). Ten microliters of the extract was added to 990 μL of ABTS^•+^ diluted in ethanol, and the resulting solution was stored at room temperature for 30 min in the dark. Absorbance was measured at 734 nm against a blank. The Trolox standard solution was used to perform the calibration curves, and the results were expressed as μmol Trolox equivalent g^−1^d.b. For quantification of total carotenoids, the supernatant was collected and transferred to a tube and taken to a Fisher Scientific glass photometric cell using acetone as a blank. The absorbance of the solution was determined at 449 nm, and Sigma-Aldrich β-carotene (St. Louis, MO, USA) was used as the standard to determine the calibration curves, and the results were expressed as mg β-carotene g^−1^d.b. according to the methodology modified by Biswas et al. (2011). Additionally, carotenoid retention percentages were determined with respect to the drying temperatures, and a simple linear regression analysis was performed from the experimental data for the pumpkin powders obtained from fresh pulp and thermally pretreated pulp using the method described by Sánchez et al. (2015).

### 2.6 Statistical analysis

Completely randomized factorial design 4^2^ with two main factors was analyzed: the type of sample (fresh pumpkin pulp and pulp with heat pretreatment) and convective drying temperatures (55 °C, 60 °C, 65 °C and 70 °C). All treatments were performed in triplicate, and the results are expressed as the means ± standard deviation (SD). The R statistical program, version 3.6.1, was used for data processing using a significance level of 0.05 (ANOVA and Tukey's test).

## 3 Results and discussion

### 3.1 Drying process

**Figure 1:**
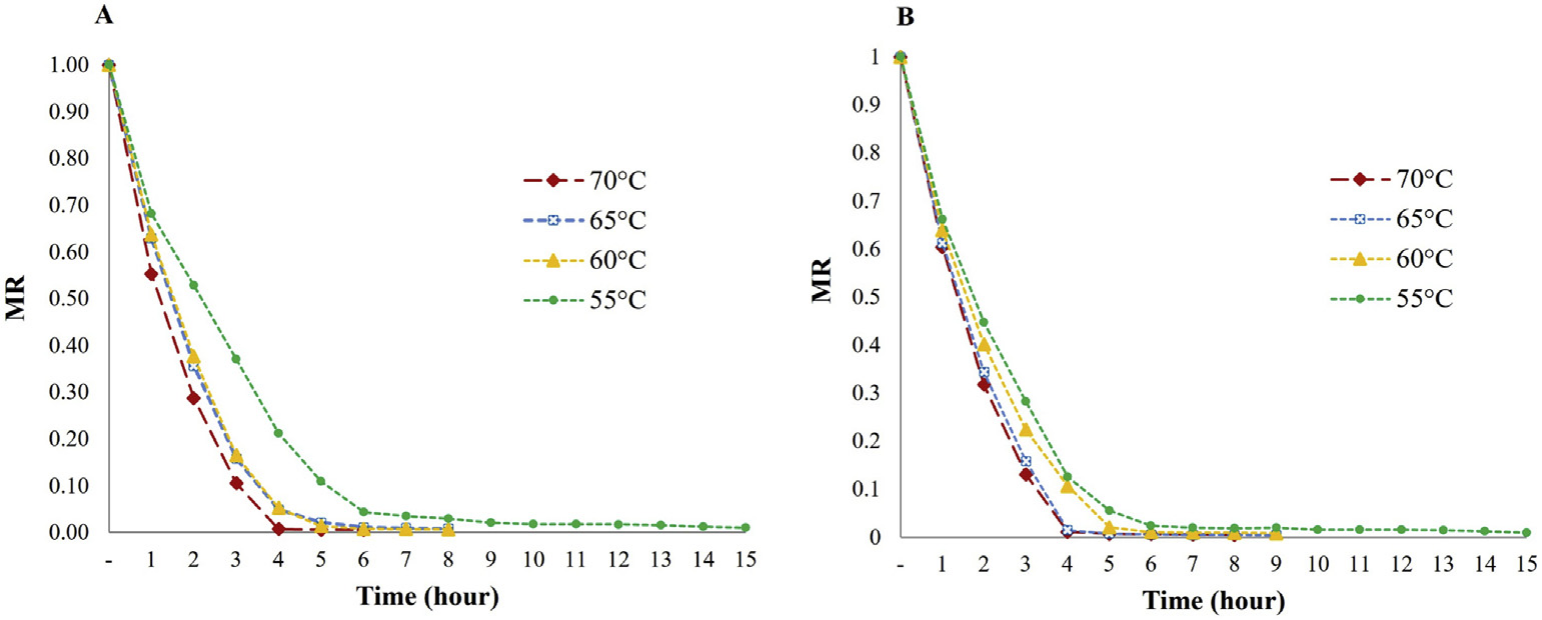
Pumpkin (*Cucurbita maxima*) convection drying at four temperatures: A) Slices of fresh pulp, B) Slices of heat pretreated pulp, n = 3.

Figure 1 shows the dimensionless moisture ratio (MR) for slices of fresh pulp and slices of heat-pretreated pumpkin pulp as a function of time for four drying temperatures.

The results in Figure 1 were strongly influenced by temperature. Drying time was significantly reduced by increasing temperature, where a higher evaporation rate is present and water loss is faster at the beginning of the process (Ireland and Mondaca, 2007; Giraldo et al., 2010; Rocha et al., 2012). The initial moisture contents were 93.07% ± 0.02 and 93.86% ± 0.01 w.b. of fresh pulp- and heat-pretreated slices, respectively, with necessary drying times of 15, 8, 7, and 6 h for temperatures of 55 °C, 60 °C, 65 °C and 70 °C, respectively (Table 3). In general, for all products, more than 80% humidity was reduced. The proportion of moisture content decreases as the drying time passes; however, for the same time, the MR may be different due to the significant effect of temperature changes and the initial conditions of pretreatment of plant material. This result is in accordance with that reported by Potosí et al. (2017), who dehydrated pumpkin (*C. moschata*) and concluded that it is more advantageous to dehydrate at higher temperatures from the point of view of energy consumption. However, at this temperature, there may be a loss of thermosensitive vitamins and other chemical compounds related to composition, aroma, and flavor characteristics.

### 3.2 Drying kinetics

Table 2 shows the parameters R^2^, $x^{2}$, and R_MSE_ of the mathematical models used in kinetically analyze drying. All models presented a good fit (R^2^ > 0.97); however, the modified Page model presented the best fit with respect to the experimental data with lower values of $x^{2}$ and R_MSE_. This result is consistent with that reported by Guiné et al. (2011), allowing us to predict the drying kinetics at four temperatures for both fresh pulp slices and heat-treated pulp for the drying constant *k*, corresponding to decreasing exponential values. A direct dependence on temperature is evident; that is, at a higher drying temperature, its value increases, and this result coincides with some studies on fruits and vegetables found by other researchers (Arslan and Özcan, 2011; Shi et al., 2019).

**Table 2:** Convective drying models of fresh and heat-pretreated slices of pumpkin pulp (*Cucurbita maxima*) at different temperatures.

Model	Variable	55 °C		60 °C		65 °C		70 °C	
		Fresh pulp	Heat pretreated pulp	Fresh pulp	Heat pretreated pulp	Fresh pulp	Heat pretreated pulp	Fresh pulp	Heat pretreated pulp
Newton	k (*h*^*−1*^)	0.00613	0.00755	0.00920	0.00843	0.00940	0.00983	0.01118	0.01028
	R^2^	0.98355	0.99245	0.98745	0.99152	0.98975	0.98685	0.99172	0.98709
	*x*^2^	0.00145	0.00061	0.00148	0.00098	0.00119	0.00154	0.00100	0.00161
	R_(MSE)_	0.03697	0.02398	0.03651	0.02974	0.03274	0.03721	0.02983	0.03785
PAGE	k (*h*^*−1*^)	0.00147	0.00290	0.00184	0.00286	0.00212	0.00211	0.00332	0.00258
	n	1.29941	1.18721	1.32681	1.21531	1.30383	1.31492	1.25493	1.28243
	R^2^	0.97443	0.99643	0.99827	0.99758	0.99894	0.99474	0.99773	0.99543
	*x*^2^	0.00237	0.00442	0.00023	0.00032	0.00014	0.00068	0.00031	0.00064
	R_(MSE)_	0.04576	0.06248	0.01355	0.01590	0.01055	0.02336	0.01562	0.02224
Modified Page	k (*h*^*−1*^)	0.00587	0.00728	0.00868	0.00807	0.00891	0.00924	0.01058	0.00965
	n	1.23218	1.18721	1.32681	1.21531	1.30383	1.31905	1.25498	1.34102
	R^2^	0.99008	0.99643	0.99827	0.99758	0.99894	0.99675	0.99773	0.99794
	*x*^2^	0.00093	0.00031	0.00023	0.00032	0.00014	0.00043	0.00031	0.00029
	R_(MSE)_	0.02871	0.01648	0.01355	0.01590	0.01055	0.01850	0.01562	0.01514
Henderson and Pabis	k (*h*^*−1*^)	0.00627	0.00769	0.00942	0.00859	0.00961	0.01004	0.01133	0.01049
	a	1.02502	1.02101	1.02956	1.02227	1.02712	1.02627	1.01714	1.02629
	R^2^	0.98414	0.99287	0.98840	0.99208	0.99056	0.98760	0.99205	0.98787
	*x*^2^	0.00149	0.00062	0.00154	0.00103	0.00123	0.00163	0.00110	0.00173
	R_(MSE)_	0.03629	0.02331	0.03510	0.02874	0.03142	0.03614	0.02922	0.03670
Modified Henderson and Pabis	k (*h*^*−1*^)	0.00413	0.00769	0.00942	0.00859	0.00518	0.00548	0.00546	0.00563
	a	-1.35747	0.00918	0.01732	0.05488	-1.43243	- 1.62605	-1.11262	-1.63614
	b	1.18378	0.50592	0.50611	0.48369	1.22484	1.32164	1.11263	1.32669
	c	1.18385	0.50592	0.50611	0.48369	1.22480	1.32162	1.11265	1.32674
	g	0.00491	0.00769	0.00942	0.00859	0.00664	0.00689	0.00757	0.00710
	h	0.00497	0.00769	0.00942	0.00859	0.00660	0.00681	0.00740	0.00705
	R^2^	0.99222	0.99287	0.98778	0.99208	0.99502	0.99324	0.99709	0.99382
	*x*^2^	0.00097	0.00084	0.00616	0.00413	0.00247	0.00341	0.00123	0.00412
	R_(MSE)_	0.02503	0.02332	0.03924	0.03213	0.02486	0.02921	0.01757	0.02620
Logarithmic	k (*h*^*−1*^)	0.00627	0.00765	0.00835	0.00778	0.00870	0.00922	0.00979	0.00952
	a	1.02323	1.02221	1.06597	1.04922	1.05647	1.05100	1.06437	1.05586
	c	-0.00762	-0.00172	-0.04561	-0.03563	-0.03687	-0.03090	-0.05621	- 0.03623
	R^2^	0.99078	0.99289	0.99227	0.99515	0.99321	0.99069	0.99593	0.99144
	*x*^2^	0.00090	0.00066	0.00130	0.00011	0.00112	0.00140	0.00086	0.00142
	R_(MSE)_	0.02725	0.02328	0.02942	0.00878	0.02737	0.03132	0.02221	0.03082
Midilli et al.	k (*h*^*−1*^)	0.00602	0.00727	0.00861	0.00798	0.00889	0.00918	0.01041	0.00960
	a	0.98307	0.99188	0.99549	0.99505	0.99687	0.99527	0.99787	0.99672
	b	0.00001	0.00001	-0.00001	-0.00001	-0.00001	-0.00001	-0.00004	-0.00001
	n	1.21005	1.21969	1.32537	1.20679	1.31201	1.31958	1.21689	1.33970
	R^2^	0.99029	0.99612	0.99826	0.98770	0.99393	0.99681	0.99766	0.99795
	*x*^2^	0.00060	0.00029	0.00035	0.00045	0.00026	0.00056	0.00061	0.00041
	R_(MSE)_	0.02144	0.01483	0.01396	0.01584	0.01147	0.01834	0.01611	0.01501

Taking the modified Page model, an activation energy of 29.47 kJmol^-1^ was found for the drying treatment of fresh pulp slices, and similar results were obtained with potato slices of 29.18 kJ mol^−1^ (Souza et al., 2019). Guiné et al. (2011), in convective drying of pumpkin pulp (*C. maxima*), reported values of 33.74 kJ mol^−1^. For heat-treated pumpkin pulp, an activation energy of 16.06 kJ mol^−1^ was less than that of fresh pulp, with a statistically significant effect (p < 0.05). This response was probably because the physical structure of the vegetable was affected by heat treatment, facilitating the water diffusion. Some studies have reported that the lower the activation energy in the drying process, the greater the diffusivity of water within the product, requiring less thermal energy in the physical transformation of liquid water (Purlis, 2019). Pretreatment is an operation that favors the movement of water towards the surface of the plant material for its evaporation, increasing the effective diffusivity coefficient and decreasing the E_a_ value (Gazor et al., 2014). This agrees with other studies, where they found E_a_ values in the pretreated samples between 21.44 kJ mol^−1^ and 28.67 kJ mol^−1^ and E_a_ between 28.21 kJ mol^−1^ and 35 kJ mol^−1^ for the samples without pretreatment (Potosí et al., 2017). Hence, structural modification due to thermal treatment in pumpkin pulp allows diminishing drying times, reducing the energy required in the process. Additionally, the exponential model for Modified Page (n) did not show significant differences (p > 0.05) regarding the drying temperature and sample condition (fresh and preheated).

### 3.3 Quality attributes of pumpkin powder

Table 3 lists the quality attributes corresponding to the physicochemical properties evaluated for each treatment. With forced convection drying at different temperatures, it was possible to obtain moisture content values between 7.10% w.b. and 8.26% w.b. and water activity (a_w_) from 0.31 to 0.34 for the powders obtained from fresh pulp; and values from 8.94% w.b. to 11.54% w.b. and water activity (a_w_) from 0.41 to 0.49 for heat-pretreated slices. Other researchers have reported moisture values between 6.06% w.b. and 10.79% w.b. for dehydrated products in *Cucurbita* varieties (Ortiz et al., 2008; Potosí et al., 2017). Pumpkin powders with low moisture content and water activity were obtained from the fresh pulp slice, shown by a significant effect (p < 0.05) in the initial conditions of the pumpkin pulp. The samples subjected to heat pretreatment show a higher moisture content, possibly because nonenzymatic browning occurred (they would caramelize the carbohydrates in the presence of sulfur amino acids), increasing hygroscopicity in powders (Joardder et al., 2017).

**Table 3:** Physico-chemical properties evaluated in pumpkin powder (*Cucurbita maxima*) obtained from slices of fresh pulp and slices of heat-pretreated pulp.

	Treatments	55 °C	60 °C	65 °C	70 °C
Moisture	Fresh pulp	8.26 ± 0.01^b^	8.31 ± 0.00^b^	7.10 ± 0.01^a^	7.39 ± 0.01^a^
	Heat Pretreated pulp	9.74 ± 0.01^c^	11.54 ± 0.01^d^	11.02 ± 0.01^d^	8.94 ± 0.01^c^
Protein	Fresh pulp	8.53 ± 0.04^a^	8.53 ± 0.03^a^	8.64 ± 0.05^a^	8.61 ± 0.07^a^
	Heat Pretreated pulp	9.41 ± 0.04^a^	9.22 ± 0.01^a^	9.27 ± 0.04^a^	9.49 ± 0.04^a^
Fat	Fresh pulp	2.17 ± 0.01^a^	2.17 ± 0.02^a^	2.18 ± 0.01^a^	2.19 ± 0.01^a^
	Heat Pretreated pulp	2.36 ± 0.02^a^	2.31 ± 0.01^a^	2.32 ± 0.02^a^	2.38 ± 0.02^a^
Ash	Fresh pulp	8.06 ± 0.06^a^	8.06 ± 0.08^a^	8.10 ± 0.08^a^	8.14 ± 0.08^a^
	Heat Pretreated pulp	7.65 ± 0.03^a^	7.49 ± 0.02^a^	7.54 ± 0.04^a^	7.71 ± 0.03^a^
Fiber	Fresh pulp	25.14 ± 0.28^a^	25.12 ± 0.30^a^	25.45 ± 0.09^a^	25.38 ± 0.30^a^
	Heat Pretreated pulp	24.53 ± 0.10^b^	24.04 ± 0.10^b^	24.18 ± 0.07^b^	24.75 ± 0.10^b^
Carbohydrates	Fresh pulp	48.83 ± 0.16^a^	48.81 ± 0.06^a^	48.44 ± 0.06^a^	48.29 ± 0.06^a^
	Heat Pretreated pulp	46.32 ± 0.04^b^	45.40 ± 0.05^b^	45.66 ± 0.04^b^	46.73 ± 0.03^b^
a_w_	Fresh pulp	0.31 ± 0.03^a^	0.34 ± 0. 15^a^	0.34 ± 0.03^a^	0.26 ± 0.02^c^
	Heat Pretreated pulp	0.42 ± 0.03^b^	0.49 ± 0.05^b^	0.47 ± 0.05^b^	0.41 ± 0.03^b^
L∗	Fresh pulp	43.70 ± 0.45^a^	49.64 ± 1.77^b^	50.83 ± 1.26^b^	51.63 ± 2.44^b^
	Heat Pretreated pulp	42.94 ± 0.36^a^	46.88 ± 2.66^b^	48.11 ± 2.58^b^	51.19 ± 2.77^b^
a∗	Fresh pulp	10.11 ± 0.25^bc^	8.64 ± 0.80^b^	5.76 ± 0.03^a^	7.33 ± 0.37^b^
	Heat Pretreated pulp	11.20 ± 0.04^c^	13.92 ± 0.61^c^	12.32 ± 1.1^c^	6.65 ± 0.23^a^
b∗	Fresh pulp	45.63 ± 0.39^b^	47.33 ± 1.14^bc^	43.59 ± 0.77^ab^	42.88 ± 1.97^a^
	Heat Pretreated pulp	44.35 ± 0.42^b^	46.72 ± 2.41^b^	44.88 ± 2.10^b^	38.26 ± 3.30^a^
ΔE	Fresh pulp	23.59 ± 0.46^ac^	21.23 ± 1.25^c^	25.33 ± 0.77^b^	24.50 ± 1.93^ab^
	Heat Pretreated pulp	24.15 ± 0.47^b^	19.00 ± 3.14^a^	20.78 ± 1.93^a^	28.30 ± 2.85^c^

Moisture, protein, fat, ash, fiber and carbohydrate content expressed in g/100 g wet basis (w.b), a_w_: water activity, L: CIE color space coordinate degree of luminosity, a^∗^: CIE color space coordinate degree of green-red, b^∗^: CIE color space coordinate degree blue-yellow and ΔE: color change, values represent means ± standard deviation, of three replications, n = 3.

The protein, fat, ash, fiber and carbohydrate contents of pumpkin powders obtained from slices of fresh and heat pretreated pulp are in accordance with those reported by the ICBF (2018). Pumpkin powders present values between 6.6-27.9 g/100 g w.b., ash (7.9–9.8 g/100 g w.b.), fat (2.5 g/100 g w.b.), fiber (6.25–24 g/100 g w.b.) and carbohydrates (48.49–58.93 g/100 g w.b.). According to Mi et al. (2012) and Enneb et al. (2020), the nutritional and compositional content depends on the variety, climatic conditions, state of maturity, processing variables, and other factors of the pumpkin fruit. A significant effect was evidenced in the thermal pretreatment applied to the pumpkin pulp slices, affecting the carbohydrate and fiber contents. This is because these macronutrients are soluble in water, especially monosaccharides, disaccharides, and soluble dietary fibers, presenting a decrease in content concerning fresh pulp pumpkin powders (Oliveira et al., 2016; Joardder et al., 2017).

Other researchers have reported values in the color coordinates in the *C. maxima* powder obtained by drying by forced convection at 70 °C for the L^∗^ coordinate of 63.38 ± 2.1, a^∗^ coordinate of 28.01 ± 3.4, and b∗ coordinate of 57.26 ± 4.8; these values differ from those found in this research, probably due to the phenotypic characteristics of the crop (Guiné and João, 2011). The CIE-L^∗^a^∗^b^∗^ coordinates of each pumpkin powder treatment did not show a significant effect on the L^∗^ value in the treatments subjected to temperatures of 60 °C, 65 °C and 70 °C. At 55 °C, a lower value in L was found, presenting a significant effect on the other treatments due to the longer exposure time in drying. Thermal pretreatment showed a significant effect in the a∗ color coordinate for drying temperatures of 60 °C, 65 °C, and 70 °C, while for the b∗ color coordinate, it was at 60 °C. The drying temperature of 70 °C presented for the color coordinates a^∗^ and b^∗^ a significant decrease in the values found with respect to the drying temperature of 55 °C, which may be related to the decrease in phytochemicals of the type carotenoids. Regarding the effects of thermal pretreatment, a significant difference was found in the total color change (ΔE), probably caused by enzymatic and nonenzymatic browning reactions, due to the participation of reducer-type carbohydrates and free amino acids (Ortiz et al., 2008; Aydin and Gocmen, 2015).

Table 4 shows the quality attributes of the techno properties evaluated in processed powders for both fresh pulp and heat-treated pulp. Values between 5.36% and 6.46% were obtained in the CWS, and no significant effect was found regarding heat treatment of the sliced pulp; in general, the values found were lower than those reported by other researchers for cassava, sweet potato, and yam powders ranging from 9% to 12% (Salcedo et al., 2017). The low percentage of carbohydrates and proteins that this type of product has and the effects of the volumetric contraction of the pores as a result of dehydration treatments could be possible reasons for this behavior (Aydin and Gocmen, 2015).

**Table 4:** Techno-functional properties of pumpkin powder (*Cucurbita maxima*) obtained from slices of fresh pulp and slices of heat-pretreated pulp.

Variable	Factor	55 °C	60 °C	65 °C	70 °C
CWS (%)	Fresh pulp	5.97 ± 0.12^a^	6.13 ± 0.14^a^	6.02 ± 0.16^a^	6.46 ± 0.10^a^
	Heat Pretreated pulp	6.01 ± 0.14^a^	5.58 ± 0.15^a^	5.36 ± 0.17^a^	5.81 ± 0.19^a^
WAC (g/g sample)	Fresh pulp	5.08 ± 0.05^b^	4.51 ± 0.06^ab^	3.42 ± 0.05^a^	5.79 ± 0.07^b^
	Heat Pretreated pulp	5.27 ± 0.06^ab^	4.85 ± 0.03^a^	6.21 ± 0.03^b^	6.52 ± 0.02^b^
OAC (g/g sample)	Fresh pulp	1.30 ± 0.05^a^	1.28 ± 0.01^a^	1.00 ± 0.04^a^	1.21 ± 0.01^a^
	Heat Pretreated pulp	1.07 ± 0.05^a^	1.04 ± 0.04^a^	1.08 ± 0.01^a^	1.01 ± 0.08^a^
BD (g/mL)	Fresh pulp	0.53 ± 0.01^a^	0.58 ± 0.01^b^	0.60 ± 0.09^b^	0.57 ± 0.02^b^
	Heat Pretreated pulp	0.64 ± 0.01^c^	0.65 ± 0.02^c^	0.57 ± 0.09^b^	0.59 ± 0.07^b^

CWS: cold water solubility, expressed as a percentage, WAC: water absorption capacity, OAC: oil absorption capacity, BD: bulk density, values represent means ± standard deviation, of three replications, n = 3.

The water absorption capacity (WAC) values were between 3.42 g/g of sample and 6.52 g/g of sample. These results are greater than those reported by Salcedo et al. (2017) in cassava, sweet potato, and yam powder, which found values between 0.89 g/g sample and 2.13 g/g sample, but similar to the value found in powder pumpkin of 5.6 ± 0.30 g/g sample (Rodríguez et al., 2020). Thus, pumpkin powder has high potential for use in food matrices such as soups, creams, sauces, and beverages due to its high water retention, where swelling and bulking can be due to the breakdown of starch granules that facilitate the formation of new hydrogen bonds with water. The oil absorption capacity (OAC) does not show a significant difference, so there was no effect of the drying temperature and the initial conditions of the pulp, either fresh or heat pretreated; however, a high oil retention capacity is reported, favoring its implementation in products with a significant participation of lipids, such as the meat industries, in comparison with cassava flour and starches, with values between 0.62 g/g sample and 0.85 g/g sample. Regarding the apparent density, values similar to those reported for yam starch diamond varieties were found (Salcedo et al., 2017).

### 3.4 Antioxidant activity by DPPH, ABTS, and total carotenoid content

**Figure 2:**
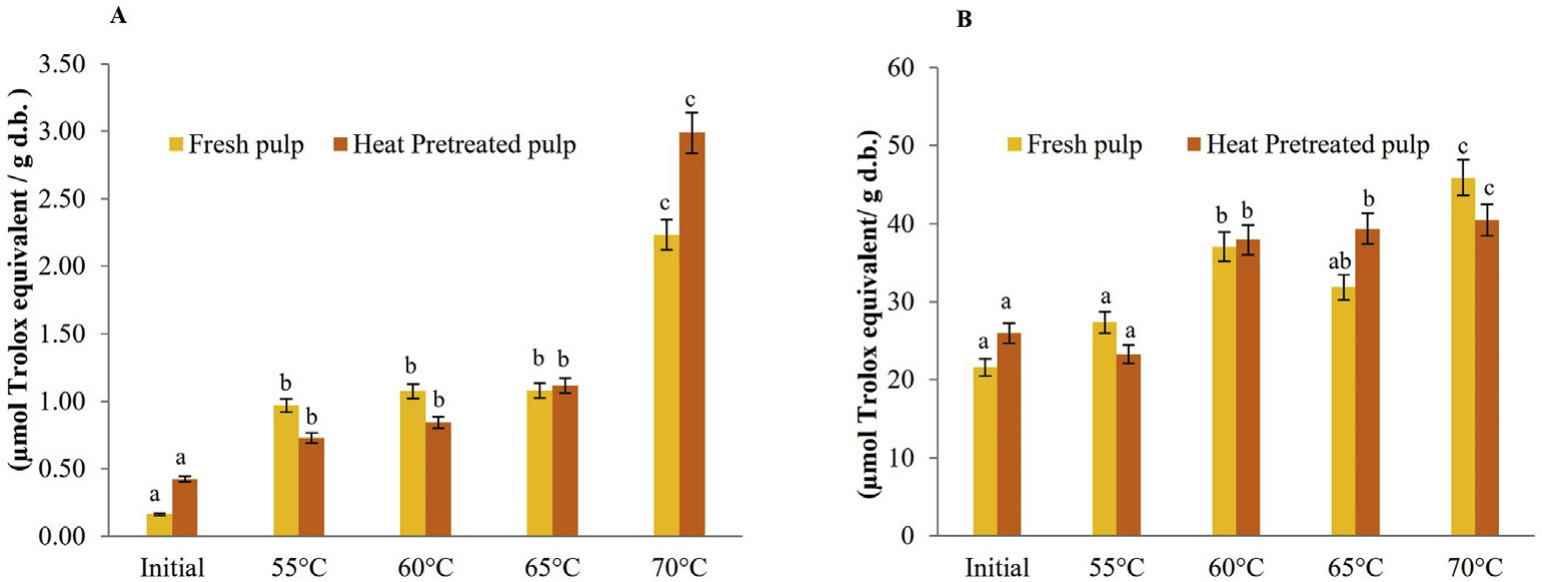
Antioxidant activity by the DPPH (A) and ABTS (B) methods evaluated in fresh and heat-pretreated pulp and in pumpkin (*Cucurbita maxima*) powders obtained by convective drying at different temperatures, n = 3.

Figure 2 shows the antioxidant activity evaluated by DPPH (Figure 2A) and ABTS (Figure 2B), presenting a significant increase in total antioxidant activity in powders obtained at higher temperatures. This was likely due to affectation in the structure of the vegetable that causes a positive effect by releasing antioxidant compounds in the matrix and facilitating their extraction. This aspect is in accordance with that reported by Silva et al. (2016). For the antioxidant capacity evaluated by the DPPH and ABTS methods, no significant difference was found for the thermally pretreated pulp and fresh pumpkin pulp (p > 0.05) factors, but there was a significant difference in temperature levels (p < 0.05).

]The antioxidant activities of the evaluated methods for DPPH and ABTS show growth with increasing temperature, where a significant difference was found for the treatments dehydrated at 70 °C concerning the lower temperatures. In the present study, antioxidant activity values of 2.99 ± 0.09 μmol Trolox equivalent g^−1^d.b. were found by DPPH of powders dried at 70 °C and ABTS of 45.92 ± 1.10 μmol Trolox equivalent g^−1^d.b. they were found. Other investigations report higher values of antioxidizing activity for freeze-dried pumpkin powders of the *C. moschata* variety, corresponding to 5.57 ± 0.02 μmol Trolox equivalent g^−1^d.b. determined by DPPH and lower values corresponding to 15.20 ± 0.50 μmol Trolox equivalent g^−1^d.b. by ABTS (Aydin and Gocmen, 2015). This may be due to the genetic behavior of the plant materials used and the sensitivity of the antioxidant action measurement methods (Re et al., 1999; Aydin and Gocmen, 2015).

**Figure 3:**
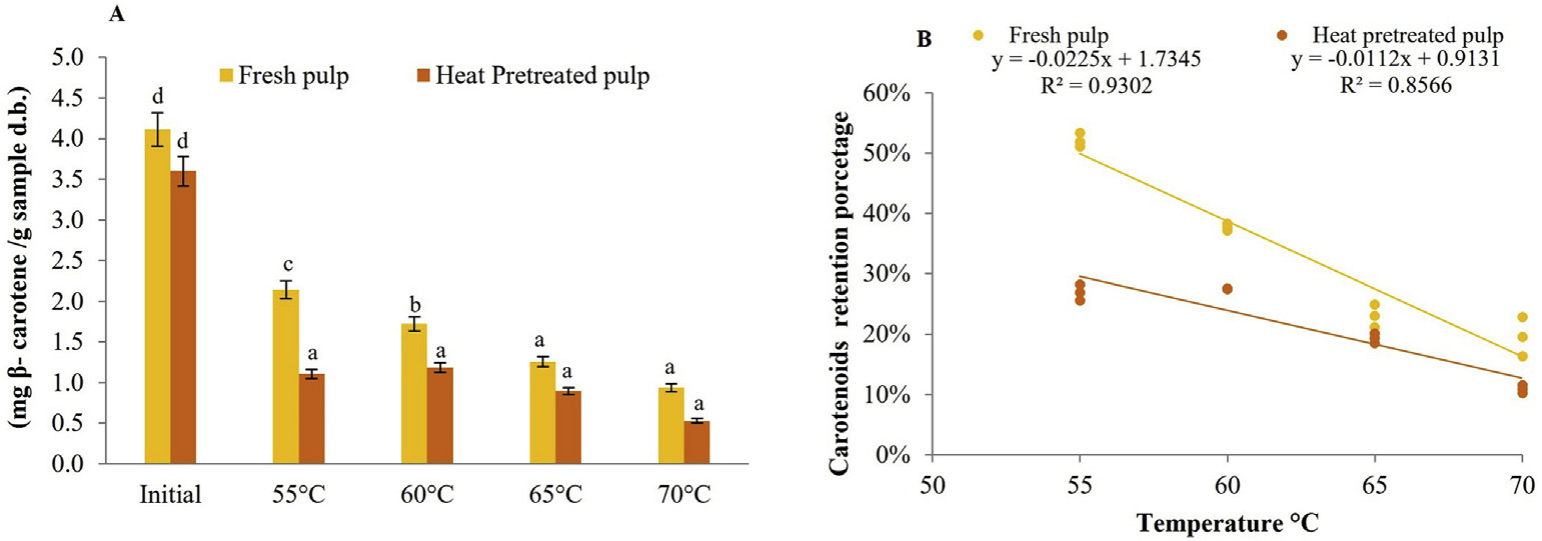
Total carotenoid content (A) and carotenoid retention potential (B) evaluated in fresh and heat-pretreated pulp and in pumpkin powders (*Cucurbita maxima*) obtained by convective drying at different temperatures, n = 3.

According to Figure 3A, the slices of fresh pulp had a concentration of 4.11 ± 1.6 mg/g d.b., while the slices of heat-treated pulp showed 3.60 ± 1.6 mg/g d.b., values similar to those reported by Jaeger et al. (2012), between 2.34 mg/g d.b. and 4.04 mg/g d.b. of total carotenoids for the *C. moschata*. For the *C. maxima* variety, values of 0.083 mg/g d.b. were reported by Zdunić et al. (2016), which are much lower than those found in the present study, most likely due to the characteristics of the soil, climate, and crop conditions, among others (Kulczynski and Gramza, 2019). For total carotenoids, a decrease is evident with respect to the fresh product due to the thermosensitivity of this bioactive compound, an aspect proposed by other researchers (Shi et al., 2013; Domínguez et al., 2015; Londoño et al., 2017). In pumpkin powder, final concentrations of total carotenoids of 1.15 mg g^-1^ d.b. and 1.42 mg g^-1^ d.b. of the *C. moschata* variety under drying conditions of 55 °C and an air velocity of 7 ms^−1^ were found by Potosí et al. (2017).

Pumpkin powders obtained from fresh pulp slices retained higher concentrations of total carotenoids (Figures 3A, B). Additionally, analysis of this variable indicated a statistically significant interaction (p < 0.05) with drying temperature and sample treatment. For the most severe dehydrating treatment corresponding to 70 °C, the powder obtained from the slices of fresh pulp presented a retention percentage of total carotenoids of 23%, while heat-pretreated pulp presented a retention percentage of total carotenoids of 15% with respect to the initial concentration and showed a degradation directly proportional to drying temperature (Topuz et al., 2011; Saini et al., 2015).

Figure 3B shows a directly proportional degradation of carotenoids with an increase in temperature, indicating almost twice the loss in retention in the samples subjected to the initial thermal pretreatment in the pumpkin pulp sheets, evidenced by the thermal sensitivity of this biocomposite. It is very probable that this loss in the pretreated treatments was caused by water leaching or chemical oxidation of this bioactive compound, although some authors indicate that this type of pretreatment before dehydration improved the retention of β-carotene during the storage of the dehydrated product, probably due to the enzyme inactivation (Oliveira et al., 2016). However, these total carotenoid retention percentages after the dehydration process in pumpkin powder, both obtained from slices of fresh pulp and thermally pretreated pulp, can be considered a product rich in total carotenoids, precursors of vitamin A (Hernández, 2014).

## 4 Conclusions

The alteration of a structure by thermal heating of pumpkin (*C. maxima*) shows a statistically significant effect in the drying process of forced convection, lowering the activation energy and facilitating moisture removal. There was a statistically significant decrease in the content of carbohydrates and fiber in the pumpkin powder samples subjected to heat pretreatment. The pumpkin powder shows high cold-water solubility, water absorption capacity, and oil absorption capacity, favoring its possible implementation in matrices for the food industry. The antioxidant activity determined both by the DPPH method and then by ABTS had a significantly increasing effect with increasing drying temperature, but the initial pretreatment of the pumpkin pulp (fresh and pretreated) did not show a significant difference. A reduction in the concentration of total carotenoids in pumpkin powders was found by the combined effect of drying temperature and prethermal processing of the samples.

## Declarations

### Author contribution statement

Carlos J. Márquez-Cardozo, Birina L. Caballero-Gutiérrez, Héctor J. Ciro-Velázquez: Conceived and designed the experiments; Performed the experiments; Analyzed and interpreted the data; Contributed reagents, materials, analysis tools or data; Wrote the paper.

Diego A. Restrepo-Molina: Analyzed and interpreted the data; Contributed reagents, materials, analysis tools or data.

### Funding statement

This work was supported by PATRIMONIO AUTÓNOMO FONDO NACIONAL DE FINANCIAMIENTO PARA LA CIENCIA Y LA INNOVACIÓN FRANCISCO JOSÉ DE CALDAS – 10.13039/100007637COLCIENCIAS (776–2017) (Contract N° 029-2018).

### Data availability statement

Data included in article/supplementary material/referenced in article.

### Declaartion of interests statement

The authors declare no conflict of interest.

### Additional information

No additional information is available for this paper.
